# Evaluation of a Sore throat Test and tReat servicE in community Pharmacies (STREP).

**DOI:** 10.23889/ijpds.v7i3.1999

**Published:** 2022-08-25

**Authors:** Samantha Turner, Haroon Ahmed, Ashley Akbari, Jackie Bethel, Rebecca Cannings-John, Andrew Evans, Efi Mantzourani

**Affiliations:** 1 Population Data Science, Swansea University; 2 Division of Population Medicine, Cardiff University; 3 Centre for Trials Research, Cardiff University; 4 Primary Care Services, Welsh Government; 5 Cardiff School of Pharmacy and Pharmaceutical Sciences, Cardiff University

## Objectives

A Sore Throat Test and Treat (STTT) service was introduced in selected community pharmacies to screen against Streptococcus A and appropriately treat infections, relieving pressure on General Practices (GP). The long-term impact on patient and NHS outcomes is unclear. A robust evaluation is required to inform future policy and roll-out.

## Approach

A matched cohort study comparing patients who received an STTT consultation in community pharmacy (exposed) compared to consultation with GP (unexposed). Individual-level data from participating STTT pharmacies were extracted from the Choose Pharmacy IT platform, anonymised and acquired into the Secure Anonymised Information Linkage (SAIL) Databank. Both cohorts were linked to other longitudinal health and administrative data within SAIL to create study outcomes (e.g. antibiotic prescribing, re-consultation, attendance/admission at Emergency Department/hospital). The impact of the STTT service was evaluated by modelling the outcomes using logistic regression to calculate odds ratios.

## Results

7553 patients exposed to the STTT service (8,313 consultations) in community pharmacies in Cwm Taf Morgannwg and Betsi Cadwaladr University Health Boards between 1st November 2018 and 28th February 2020, were successfully anonymised and acquired into the SAIL Databank (97.16%). 99.5% of these individuals were then linked to the Welsh Demographic Service Dataset (WDSD), enabling demographics to be generated. 6,665 distinct consultations remained in the exposed group after exclusion criteria were applied. A matched unexposed cohort will be created, and odds ratios will be presented to compare the risk of each outcome for those exposed to the STTT service compared to usual care.

## Conclusion

This innovative study is the first to link national pharmacy service data to individual-level population-scale longitudinal health and administrative data in the SAIL Databank. Our findings will inform clear and actionable recommendations regarding the service design and future roll-out of STTT services across the UK and internationally.

